# loco-pipe: an automated pipeline for population genomics with low-coverage whole-genome sequencing

**DOI:** 10.1093/bioadv/vbae098

**Published:** 2024-07-11

**Authors:** Zehua T Zhou, Gregory L Owens, Wesley A Larson, Runyang Nicolas Lou, Peter H Sudmant

**Affiliations:** Department of Integrative Biology, University of California Berkeley, Berkeley, CA 94720, USA; Department of Biology, University of Victoria, Victoria, BC V8P 5C2, Canada; National Marine Fisheries Service, Alaska Fisheries Science Center, National Oceanographic and Atmospheric Administration, Auke Bay Laboratories, Juneau, AK 99801, USA; Department of Integrative Biology, University of California Berkeley, Berkeley, CA 94720, USA; Department of Integrative Biology, University of California Berkeley, Berkeley, CA 94720, USA; Center for Computational Biology, University of California Berkeley, Berkeley, CA 94720, USA

## Abstract

**Summary:**

We developed loco-pipe, a Snakemake pipeline that seamlessly streamlines a set of essential population genomic analyses for low-coverage whole genome sequencing (lcWGS) data. loco-pipe is highly automated, easily customizable, massively parallelized, and thus is a valuable tool for both new and experienced users of lcWGS.

**Availability and implementation:**

loco-pipe is published under the GPLv3. It is freely available on GitHub (github.com/sudmantlab/loco-pipe) and archived on Zenodo (doi.org/10.5281/zenodo.10425920).

## 1 Introduction

Low-coverage whole genome sequencing (lcWGS) is a cost-effective approach to characterize patterns of genetic variation across entire genomes (Lou *et al.* 2021). It has become increasingly popular for both addressing basic evolutionary questions and solving applied biodiversity conservation problems (Clucas *et al.* 2019; Therkildsen *et al.* 2019; Mérot *et al.* 2021; Ulmo-Diaz *et al.* 2023). Because individual genotypes cannot be confidently assigned from low sequencing depth, lcWGS relies on a suite of specialized software programs that account for genotype uncertainty using a probabilistic framework (Nielsen *et al.* 2012, 2011; Korneliussen *et al.* 2014). These software programs can present a steep learning curve for beginners as they have complex workflows with intricate dependencies and require substantial background knowledge in programing, population genetics, and statistics. Even for experienced users, manually running these software programs can be labor intensive and error prone (Lou and Therkildsen 2022; Dallaire *et al.* 2023). Several workflow management systems, including Snakemake (Mölder *et al.* 2021) and Nextflow (Di Tommaso *et al.* 2017), present great opportunities to automate complex computational workflows and vastly increase their accessibility, reproducibility, and efficiency. They have been successfully employed in automated computational pipelines including grenepipe (Czech and Exposito-Alonso 2022) and snpArcher (Mirchandani *et al.* 2024) for the analyses of high coverage sequencing data. However, automated lcWGS pipelines that can account for genotype uncertainty have not yet been made available [but see ANGSD-wrapper (Durvasula *et al.* 2016), https://github.com/clairemerot/angsd_pipeline, and https://github.com/therkildsen-lab/genomic-data-analysis for collections of scripts for lcWGS data analysis, and PoolParty2 (Willis *et al.* 2023) for a pipeline that implements a pool-seq approach to lcWGS data].

Here, we present loco-pipe (**lo**w-**co**verage **pipe**line), an automated Snakemake pipeline that streamlines a set of commonly used population genomic analyses for lcWGS data which can be launched with a single line of code. loco-pipe incorporates several best practices and filtering steps (Korneliussen *et al.* 2014; Lou *et al.* 2021; Mérot *et al.* 2021) and is easily customizable. loco-pipe is highly parallelizable, integrates with a software package manager, and outputs results in a well-defined structure. We provide users with comprehensive documentation, including extensive in-line annotation, a README file with general instructions, and a user’s manual containing detailed descriptions of each step of the pipeline. In addition, we provide a quick start guide with an example dataset, with which users can quickly test and visualize results. loco-pipe is available on GitHub, and we welcome contributions.

## 2 Pipeline overview

### 2.1 Pipeline preparation

Before analyses can be conducted, several key input files must be prepared. Users provide: (i) a reference genome sequence in fasta format; (ii) sequence alignment files in BAM format; (iii) a sample table; and (iv) a chromosome table. Users must also edit the configuration file to select which analyses they would like to run and to modify the relevant parameters. The pipeline is then launched with a single command.

### 2.2 Sequencing depth calculation and depth filter determination

**Figure 1.**
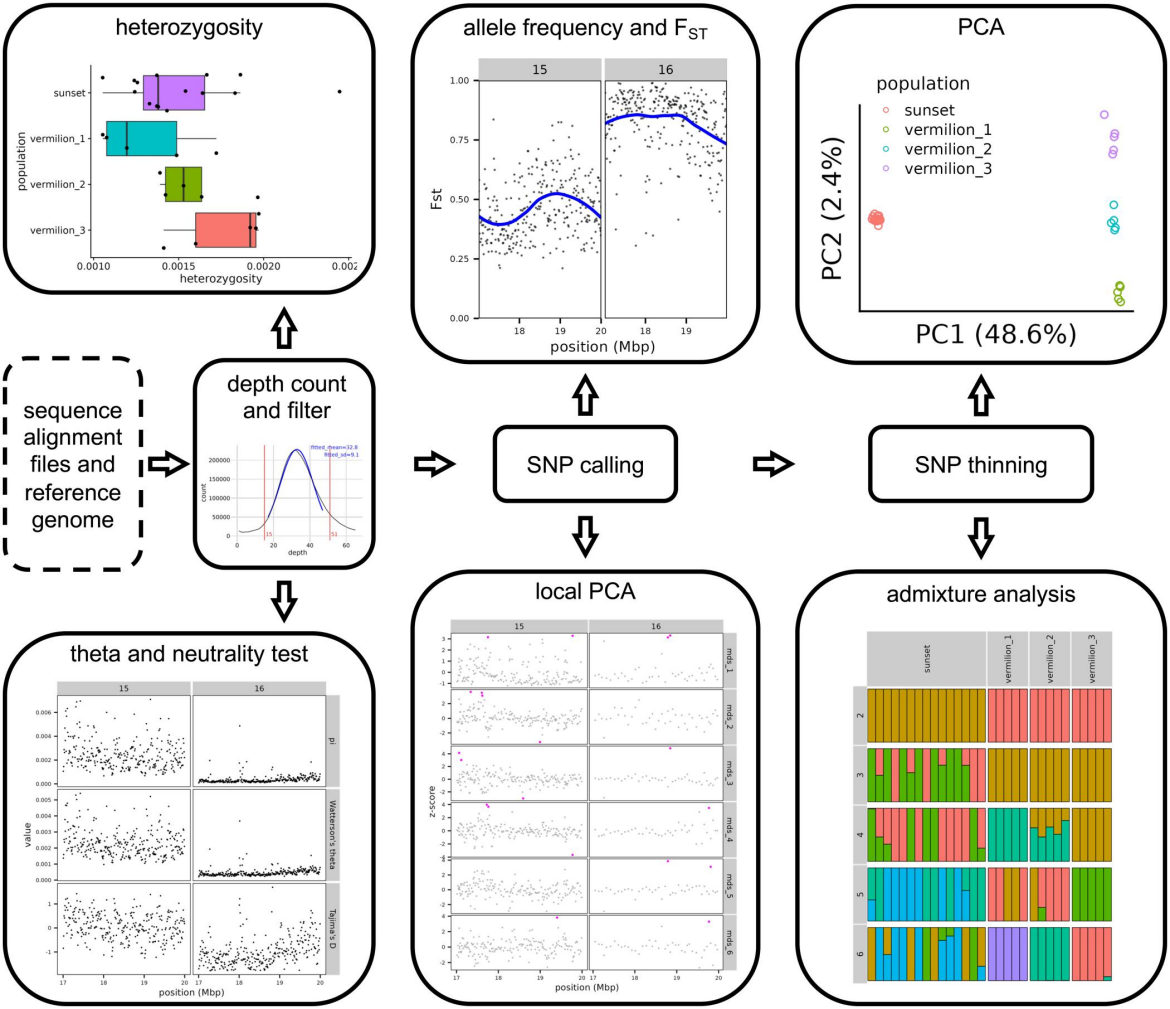
A simplified flowchart of loco-pipe highlighting its key functionalities. The box in dotted lines represents user-provided input files, and boxes in solid lines represent key analytical steps in the pipeline. Plots are generated using our example dataset.

loco-pipe first calculates the sequencing depth at each site summed across all samples with ANGSD (Korneliussen *et al.* 2014). We fit a truncated normal distribution to the bulk of the empirical depth distribution, excluding both tails. Users can then set a depth filter for subsequent analyses in standard deviations from the mean. The empirical distribution, fitted normal distribution, and filters are automatically plotted for the user (Fig. 1).

### 2.3 SNP calling and thinning

loco-pipe uses ANGSD to identify single nucleotide polymorphisms (SNPs) across the genome. Depth filters (determined in the previous step) and other commonly used filters (e.g. removal of multiallelic sites) are automatically applied, alongside any additional user-specified filters (e.g. minor allele frequency thresholds, a list of sites that analyses should be restricted on, etc). The positions of all SNPs, as well as the major and minor alleles, are stored and indexed to be used in downstream analyses. Genotype likelihoods are also output in beagle format (Browning *et al.* 2018).

Several population genetic analyses assume a set of independent (i.e. unlinked) SNPs. Additionally, some analyses are computationally prohibitive to run across the entire genome. To address this, we implement SNP thinning to retain one out of every n SNPs (n is a user-defined parameter). This approach is an efficient and easily automatable alternative compared to the more sophisticated linkage disequilibrium (LD) pruning methods.

### 2.4 Global PCA and admixture analyses

Principal component analysis (PCA) and admixture analysis are both incorporated into loco-pipe to characterize genome-wide patterns of population structure. Both analyses can use the thinned beagle file as their input by default. PCA is implemented using PCAngsd (Meisner and Albrechtsen 2018) and outputs plots of individuals projected along principal component axes (Fig. 1). Admixture analysis is implemented using Ohana (Cheng *et al.* 2017). loco-pipe automatically converts the beagle file into the required input format, estimates individual admixture proportions and allele frequencies assuming k source populations (range of k set by user), and outputs an admixture plot (Fig. 1).

### 2.5 Population-specific PCA and admixture, allele frequencies, and Fst

The aforementioned analyses are conducted across all samples; however, it is often useful to perform population genetic analyses on predefined groupings of individuals. loco-pipe enables users to define sample groupings and estimate population-specific allele frequencies, sample allele frequency (SAF) likelihoods, and site frequency spectrum (SFS). The population-specific SFSs are also plotted by the pipeline, along with the expected SFS in a neutrally evolving population for the ease of comparison and troubleshooting. For each population pair, loco-pipe outputs genome-wide per-site Fst estimates and Manhattan plots (Fig. 1). These Fst estimates are calculated using several ANGSD functions which loco-pipe automates. PCA and admixture analyses can also be performed on individual populations.

### 2.6 Genetic diversity estimates

To estimate genetic diversity from lcWGS, it is critical to evaluate both variable and invariable sites. loco-pipe identifies all sites passing depth and quality filters and uses these to calculate different estimators of θ (theta) and neutrality statistics for each population per SNP and in windows. Estimates of π, Watterson’s θ, and Tajima’s D in sliding windows are plotted for each population separately (Fig. 1). loco-pipe outputs these statistics by automating several ANGSD functions. To obtain estimates of heterozygosity for each individual, loco-pipe uses a similar workflow as above and visualizes its distribution in each population with a box plot (Fig. 1).

### 2.7 Local PCA

Local PCA is a powerful method to identify regions of the genome exhibiting distinctive patterns of variation (Li and Ralph 2019). This approach can identify regions under selection or associated with structural variants and other genomic features (Li and Ralph 2019; Todesco *et al.* 2020; Harringmeyer and Hoekstra 2022). Local PCA is implemented using the R package lostruct (Li and Ralph 2019). However, because lostruct is not designed to work on genotype likelihoods, loco-pipe implements a custom set of computational analyses enabling local PCA with low-coverage data. Briefly, loco-pipe performs windowed PCA on genotype likelihoods using PCAngsd, threads these PCAs into lostruct to calculate a distance matrix between windows, performs multidimensional scaling (MDS), and finally plots the result (Fig. 1).

## 3 Major advantages of loco-pipe

loco-pipe provides user-friendly access to many complex population genetic methods. For those inexperienced with population genetics and low-coverage data analysis, loco-pipe incorporates best practices and helps avoid common pitfalls (Korneliussen *et al.* 2014; Lou *et al.* 2021; Mérot *et al.* 2021). For example, using loco-pipe ensures that both variable and invariable sites are included in analyses of diversity, and that the frequencies of the same allele at a given locus are estimated in different populations so that the values are comparable across populations. loco-pipe also automatically applies several commonly used quality filters, depth filters, and accounts for the effect of LD by default. loco-pipe also makes it easy to sanity check important intermediate outputs (e.g. read depth distribution, the SFS), which is essential for spotting technical artifacts that can impact downstream analyses. Lastly, loco-pipe uses conda as a built-in package manager automating software installation and ensuring compatibility.

**Figure 2.**
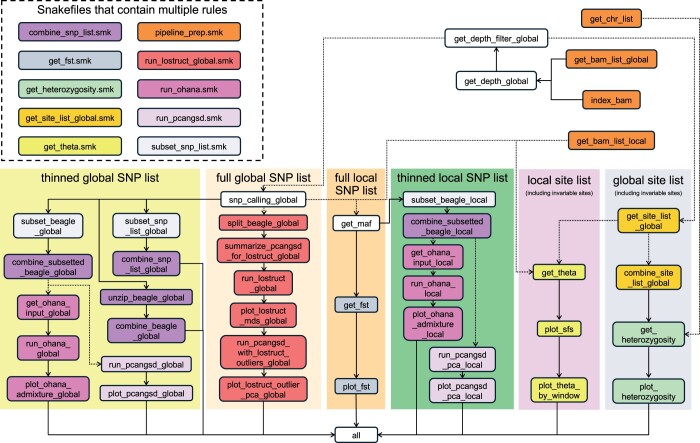
The full flowchart of loco-pipe. Each box represents a Snakemake rule and is colored based on the major groups of analyses in the form of separate Snakefiles (shown at the top left corner). The differently colored shades indicate the types of SNPs or sites on which the analyses are conducted. Dashed arrows indicate key modules that can be turned on or off in the configuration file. The four orange boxes at the top right corner (part of the “pipeline_prep.smk” Snakefile) are the starting points of the pipeline, and the “all” box at the bottom is the end point.

For experienced users, the automation and parallelization offered by loco-pipe provide a standardized pipeline to assess large datasets, tune parameters, and troubleshoot. Implemented in Snakemake, loco-pipe is able to pick up where it left off in the case of any errors or hardware failures. Most adjustments to the pipeline can be made by editing the configuration file. loco-pipe is also highly modular, making it convenient to turn off part of the pipeline or to integrate new modules (Fig. 2). We encourage pull requests from the community to increase its functionality.

We provide many resources for users to learn loco-pipe. All loco-pipe code is documented with extensive in-line annotation and a user’s manual. A tutorial with detailed instructions on how to set up, customize, and launch the pipeline is also available. This tutorial includes a small dataset as a representative example of typical lcWGS data. It consists of 30 individuals of two closely related rockfish species: sunset and vermilion (Fig. 1). Two 3Mbp regions of two chromosomes were selected such that the entire pipeline runs to completion in just a few minutes. We strongly advise users to take advantage of these resources to gain a good understanding of the mechanisms of loco-pipe (and lcWGS more broadly) instead of using it as a black box, which can lead to spurious results and erroneous interpretations. We welcome feedback, bug reports, and feature requests on the loco-pipe GitHub page.

## Data Availability

The code for loco-pipe and the test dataset are freely available on GitHub at github.com/sudmantlab/loco-pipe.
